# Content validity of the tinnitus outcome questionnaire for sound management

**DOI:** 10.1371/journal.pone.0251244

**Published:** 2021-05-06

**Authors:** Hye Yoon Seol, Ga-Young Kim, Mini Jo, Soojin Kang, Young Sang Cho, Sung Hwa Hong, Il Joon Moon

**Affiliations:** 1 Hearing Research Laboratory, Samsung Medical Center, Seoul, Korea; 2 Medical Research Institute, Sungkyunkwan University School of Medicine, Suwon, Korea; 3 Department of Otorhinolaryngology-Head and Neck Surgery, Samsung Medical Center, Sungkyunkwan University School of Medicine, Seoul, Korea; 4 Department of Otorhinolaryngology-Head and Neck Surgery, Samsung Changwon Hospital, Sungkyunkwan University School of Medicine, Changwon, Korea; University of California, Los Angeles, UNITED STATES

## Abstract

Standardized instruments are often used to monitor one’s progress in tinnitus relief although they were developed to screen and diagnose tinnitus. The need for the development for a tinnitus outcome assessment tool is high in the field of audiology and otolaryngology. The purpose of this study was to develop a tinnitus outcome questionnaire for sound management (listening to sound stimuli for tinnitus relief) and assess its content validity. A total of 32 questions with six domains (Tinnitus characteristics, the impact of tinnitus, tinnitus and hearing issues, handedness, tinnitus management, and sound management outcome) were generated after closely investigating major tinnitus questionnaires used worldwide (i.e. Tinnitus Handicap Inventory and Tinnitus Handicap Questionnaire) as well as literature. Ten healthcare professionals evaluated the appropriateness of the questionnaire items on a five-point Likert scale, where 1 is strongly inappropriate and 5 is strongly appropriate. Content relevance was assessed by computing the content validity index with the cut-off value of 0.75. Each response was first weighted as follows: 1 = 0; 2 = 0.25; 3 = 0.5; 4 = 0.75; and 5 = 1.0. The weighted average was then calculated. Items with a content validity index less than 0.75 were discarded and some items were revised according to the experts’ feedback. As a result, 31 out of the 32 items had the content validity index higher than 0.75, indicating that the items are appropriate to obtain information about the six domains. Reflecting the experts’ feedback, some questions were revised to be more specific. The study provides a baseline structure regarding potential questions to be included in a tinnitus outcome questionnaire for sound management. Development and standardization of such questionnaire would be a pathway to validating tinnitus relief via sound therapy.

## Introduction

Tinnitus, commonly known as “ringing” in the ears, refers to phantom sounds in the ear [1]. Numerous literatures have reported various etiologies of tinnitus: otologic and neurologic disorders (i.e. hearing loss, otitis media, ear wax, head injury etc.), drugs (i.e. anti-inflammatory drugs, chemotherapy agents etc.) and anatomical issues (i.e. tensor tympani spasm, patulous eustachian tube etc.) [2, 3]. Along with the etiologies, tinnitus expresses a wide variety of characteristics in terms of onset, severity, laterality, frequency, and types of sound. It can occur abruptly or progressively and it can be constant or intermittent. For types of sound, tinnitus includes, but not limited to, buzzing, puretone, music, and hissing sounds. Tinnitus associated comorbidities have been well researched [4, 5]. For instance, one might experience non-bothersome tinnitus only at night in his or her own quiet bedroom while others might experience bothersome ringing to the point of feeling suicidal. Studies have also reported a high prevalence of tinnitus [6, 7]. Bhatt et al. performed a cross-sectional analysis on 75,764 respondents from the 2007 National Health Interview Survey and reported that approximately one in ten adults experience tinnitus in the United States [7]. A high prevalence of tinnitus with increasing age in South Korea was reported by Lee et al. who performed a big data analysis from the National Health Information database. In 2015, 0.78 million per 51 million sought medical care regarding tinnitus [6].

There are several approaches for tinnitus management: sound amplification, surgery, tinnitus retraining or cognitive behavioral therapy, magnetic stimulation, and pharmaceuticals [8]. Sound amplification refers to hearing technology, such as hearing aids, as well as background noise and noise maskers [8]. A combination of counselling and ear-level noise generators can be utilized to separate tinnitus from one’s negative thoughts and responses [8]. This approach is called tinnitus retraining therapy. Cognitive behavioral therapy, on the other hand, involves counselling and relaxation techniques to correct one’s negative thoughts and responses towards tinnitus [8]. An appropriate approach is determined by healthcare professionals based on the patient’s complaints, possible underlying condition, and characteristics of tinnitus.

To monitor one’s progress in tinnitus relief, several standardized instruments are utilized, such as the Tinnitus Handicap Inventory (THI) and Tinnitus Handicap Questionnaire (THQ) [9]. The Korean version of the THI shown to have good reliability and validity, is most widely used in Korea [9, 10]. However, it is important to note that these standardized questionnaires were not developed to measure treatment outcome. In the case of THI, it was originally developed for the purpose of screening and diagnosing tinnitus, but it is frequently utilized to examine the effectiveness of tinnitus treatments [11]. Langguth et al. adds that there is need for the development of an outcome measurement tool to monitor treatment effects [12]. A more recent study in 2017 reports that clinical trials regarding tinnitus have low methodological and reporting quality [13] and this lack of outcome assessment tool also partly contributes to the difficulty finding a “cure” for tinnitus [14]. The purpose of this study is to design a tinnitus outcome questionnaire, specifically for sound management as a new outcome measure and assess its content validity.

## Materials and methods

### Phase I: Generating questionnaire items

Phase I involved generating questionnaire items by investigating widely accepted standardized tinnitus questionnaires, such as THI, THQ, Tinnitus Functional Index (TFI), and Tinnitus Sample Case History Questionnaire (TSCHQ) [12, 15–17], and literature on tinnitus and management using sound [18]. Most standardized questionnaires contain a wide variety of domains including social, mental, and physical functioning, affective responses towards tinnitus, and outcome measurements [12, 17, 19]. Studies regarding sound management for tinnitus relief used the visual analogue scale (VAS) to measure annoyance, loudness, awareness caused by tinnitus [20, 21]. The questionnaire items were created based on the main purposes of the tinnitus outcome questionnaire for sound management: quantifying tinnitus characteristics and the improvement of tinnitus before and after listening to sound stimuli. Different domains were created upon examination of some standardized questionnaires to quantify tinnitus characteristics and the impact of tinnitus on individuals’ lives. For instance, Tinnitus Questionnaire had three categories: sleep disturbance, emotional distress, and auditory perceptual difficulties [19]. The auditory perceptual difficulties domain included items related to hearing issues. Thus, the authors added a domain of tinnitus and hearing issues in the questionnaire. As a result, a total of 32 questions with six categories were generated: tinnitus characteristics, the impact of tinnitus, tinnitus and hearing issues, handedness, tinnitus management, and sound management outcome.

### Phase II: Assessing the content validity of the items

Phase II entailed content validation, which is “determination of content representativeness or content relevance of the elements or items of an instrument” according to Lynn 1986 [22]. Once all items were generated, an online anonymous survey was created via Google Forms and was distributed to ten healthcare professionals. The professionals were recruited from January 2020 to November 2020 from the following inclusion criteria: (a) adults 19 years old or older, (b) practitioners in the field of audiology or otolaryngology, and (c) native speakers of Korean. Exclusion criteria included individuals who were not willing to provide informed consent. All experimental procedures were approved by the regulations set by Samsung Medical Center’s Institutional Review Board and were carried out in accordance with approved guidelines. The experts were first informed about the purpose, risks and benefits, voluntary nature, and consequences of withdrawal of the survey. Verbal consent was obtained from all experts. The mean age of the professionals was 38.2 years (SD = 2.9). Average years of overall clinical experience and clinical experience in tinnitus were 11.1 (SD = 3.8) and 7.4 years (SD = 2.2), respectively. Demographics of the experts are described in Table 1. The experts judged how appropriate each question is to collect information regarding tinnitus characteristics and the effect of sound management on a five-point Likert scale, where 1 is strongly inappropriate and 5 is strongly appropriate. The experts were also requested to provide feedback, especially when they evaluated the item “1-strongly irrelevant” and “2-irrelevant” so that the authors could revise the items. Upon completion, the content validity index (CVI) with a cut-off value of 0.75, which was recommended in multiple studies, was calculated to validate the content of the questionnaire items [23, 24]. As the CVI is an index of inter-rater agreement, there is as an issue of chance agreement which Polit et al. tried to address utilizing the kappa statistic [25]. Similar to findings in Cicchetti et al., the value of 0.75 was found to be “excellent” with 10 or more experts [25, 26]. Thus, 0.75 was selected to be the cut-off value for this study. To compute the CVI, each response was first weighted as follows: 1 = 0; 2 = 0.25; 3 = 0.5; 4 = 0.75; and 5 = 1.0 and the average was calculated. As the cut-off value for the CVI is 0.75, items with a CVI less than 0.75 were considered to be less relevant, and therefore, discarded. No statistical analysis was performed as it was not necessary for the study. Several items were revised according to the experts’ feedback.

**Table 1:** Demographics of the healthcare professionals (n = 10).

No.	Age (yo)	Sex (M/F)	Clinical experience (yrs)		Highest education	Job setting
			Overall	Tinnitus		
1	39	M	16	6	Doctorate	Hospital
2	40	M	11	11	Doctorate	Hospital
3	40	M	12	6	Master’s	Hospital
4	42	M	18	10	Doctorate	Hospital
5	37	F	9	5	Master’s	Hospital
6	33	M	10	5	Master’s	Hospital
7	39	F	9	9	Doctorate	Hospital
8	35	F	5	5	Doctorate	University
9	42	M	14	10	Doctorate	Hospital
10	35	M	7	7	Master’s	Hospital

## Results

### Tinnitus outcome questionnaire for sound management

As described above, six domains and 32 questions were formed for the tinnitus management outcome questionnaire for sound management. The ‘Tinnitus characteristics’ domain consists of questions regarding the onset, cause, loudness, annoyance, and frequency of tinnitus. Items in ‘The impact of tinnitus’ include awareness of and stress caused by tinnitus. Items related to hyperacusis and hearing loss are in ‘Tinnitus and hearing issues’. The ‘Handedness’ domain asks whether one is right- or left-handed. In the ‘Tinnitus management outcome’, individuals are asked to report various methods they have tried and the effectiveness of each method in relieving tinnitus. Lastly, the ‘Sound management outcome’ involves the loudness and annoyance of tinnitus, changes in awareness or reaction to tinnitus after listening to sound stimuli, and other stimuli that individuals wish to listen to for tinnitus relief.

### Assessing content validity of the questionnaire

CVIs were calculated for each item and are shown in Table 2. Out of the 32 items, only one question (*How was the onset of tinnitus*?) had a CVI of 0.73 which is less than the cut-off of 0.75. Thus, the question is considered to be less relevant and discarded from the questionnaire. 11 out of 32 items had the highest CVI which was 0.98 *(Does stress cause tinnitus or worsen your tinnitus*?*; Have you received a hearing test and been diagnosed with hearing loss*?*; What have you tried to relieve your tinnitus*?*; How long have you tried the methods you selected above*?*; Was if effective*? *If so*, *how much did your tinnitus improve*?*; Are you currently wearing hearing aids or have you used hearing aids in the past*?*; How long have you been wearing your hearing aids or how long have you used your hearing aids*?*; How many hours in a day do you wear your hearing aids or how many hours in a day have you worn your device*?*; Which environments do you mostly wear your hearing aids or which environments have you mostly used your hearing aids*?*; Are there any changes in awareness or reaction to your tinnitus after listening to sound stimuli for N weeks*?; and *How annoying is your tinnitus now*?). Four items *(Which ear do you experience tinnitus*?*; When did you start experiencing tinnitus*?*; How annoying is your tinnitus now*?; and *Do you feel that certain sounds others hear as quiet or comfortable are loud to you*?*)* had the CVI of 0.95. Six questions had the CVI of 0.93: *Do you constantly experience tinnitus*?*; How much are you aware of your tinnitus*?*; Are you currently taking any medications due to your tinnitus*?*; If yes*, *what medications are you currently taking*?*; Which ear do you experience tinnitus*?; and *How loud is your tinnitus right now*?. One question (*What does your tinnitus sound like*?) had a CVI of 0.90. Lastly, the rest of the items had a CVI below 0.90 ranging between 0.78 and 0.89. These items include: *Which ear are you wearing your device or which ear did you wear the device*?*; If you experience tinnitus in both ears*, *which ear is experiencing more severe tinnitus*?*; How loud is your tinnitus right now*?*; How frequently do you experience tinnitus*?*; Are you right- or left-handed*?*; If not*, *do you experience difficulty hearing*?*; Are there any other sound stimuli you wish to listen to*? *If so*, *what are they*?*; What is the cause of your tinnitus*?; and *If yes*, *does your medication worsen your tinnitus*?

**Table 2:** Tinnitus outcome questionnaire for sound management and CVIs for each item.

Domain	No.	Question	CVI
**Tinnitus characteristics**	1	Which ear do you experience tinnitus?	**0.95**
	2	When did you start experiencing tinnitus?	**0.95**
	3	If you experience tinnitus in both ears, which ear is experiencing more severe tinnitus?	**0.88**
	4	How frequently do you experience tinnitus?	**0.85**
	5	What is the cause of your tinnitus?	**0.80**
	6	How was the onset of your tinnitus?	0.73
	7	What does your tinnitus sound like?	**0.90**
	8	Do you constantly experience tinnitus?	**0.93**
	9	How loud is your tinnitus right now? (0-not at all, 10-extremly loud)	**0.88**
	10	How annoying is your tinnitus now? (0-not at all, 10-extremly annoying)	**0.95**
**The impact of tinnitus**	11	How much are you aware of your tinnitus?	**0.93**
	12	Does stress cause tinnitus or worsen your tinnitus?	**0.98**
**Tinnitus and hearing issues**	13	Have you received a hearing test and been diagnosed with hearing loss?	**0.98**
	14	If not, do you experience difficulty hearing?	**0.83**
	15	Do you feel that certain sounds others hear as quiet or comfortable are loud to you?	**0.95**
**Handedness**	16	Are you right- or left- handed?	**0.85**
**Tinnitus management**	17	Are you currently taking any medications due to your tinnitus?	**0.93**
	18	If yes, what medications are you currently taking?	**0.93**
	19	If yes, do your medications worsen your tinnitus?	**0.78**
	20	What have you tried to relieve your tinnitus?	**0.98**
	21	How long have you tried the methods you selected above?	**0.98**
	22	Was it effective? If so, how much did your tinnitus improve?	**0.98**
	23	Are you currently wearing hearing aids or have you used hearing aids in the past?	**0.98**
	24	Which ear are you wearing your device or which ear did you wear the device?	**0.89**
	25	How long have you been wearing your hearing aids or how long have you used your hearing aids?	**0.98**
	26	How many hours in a day do you wear your hearing aids or how many hours in a day have you worn your device?	**0.98**
	27	Which environments do you mostly use your hearing aids or which environments have you mostly used your hearing aids?	**0.98**
**Sound management outcome**	28	Are there any changes in awareness or reaction to your tinnitus after listening to sound stimuli for N weeks?	**0.98**
	29	Which ear do you experience your tinnitus?	**0.93**
	30	How loud is your tinnitus now? (0-not at all, 10-extremly loud)	**0.93**
	31	How annoying is your tinnitus now? (0-not at all, 10-extremly annoying)	**0.98**
	32	Are there any other sound stimuli you wish to listen to? If so, what are they?	**0.83**

## Discussion

Along with hearing loss, tinnitus has become a major health concern affecting various aspects of individuals’ lives [27, 28]. However, it is often difficult to manage tinnitus due to the lack of objective examination and consensus regarding the pathophysiology of tinnitus, and limited options for management [13, 28]. Clinical evaluations involving case history, audiometry, and tinnitogram could provide an insight as to one’s tinnitus characteristics and suggest possible intervention methods as an attempt to reduce tinnitus. However, when it comes to the efficacy of the intervention, healthcare professional typically rely heavily on self-report questionnaires [29]. Even these questionnaires have a limited capability of measuring tinnitus treatment outcome as their main purpose is diagnosing tinnitus [12, 27]. Thus, developing an outcome questionnaire assessing changes in tinnitus before and after intervention is high in demand in clinical practice as well as research.

In this study, the authors designed a comprehensive tinnitus outcome questionnaire which has general items about tinnitus characteristics as well as items specific to sound management. The authors assessed the validity of its content which is the first step when developing questionnaires examining treatment effects [29]. The questionnaire items were created upon reviewing standardized tinnitus questionnaires and research studies. Ten healthcare professionals with clinical experience in tinnitus assessed the content validity. Among 32 items, 31 items illustrated that their content representativeness is appropriate with the CVI over 0.75 which is its cut-off value. In ‘Tinnitus characteristics,’ the experts evaluated the following three questions as the most relevant questions in the section: *Which ear do you experience tinnitus*?*; When did you start experiencing tinnitus*?; and *How annoying is your tinnitus right now*?. These questions are available in many questionnaires that are already in use (THI, TFI, THQ, and TSCHQ). One question (*How was the onset of tinnitus*?) had the lowest CVI (0.73) overall with the experts’ feedback of vagueness of the question and this item was removed. In the “The impact of tinnitus” section, all questions (*How much are you aware of your tinnitus*? and *Does stress cause tinnitus or worsen your tinnitus*?) had higher CVI values of 0.93 and 0.98, respectively. In the ‘Tinnitus and hearing issues’ domain, two out of three questions had a CVI higher than 0.95 and one question asking if one thinks to have hearing loss had a CVI of 0.83. Experts suggested the following phrase to be included in the question: *If not*, *do you experience difficulty hearing*? and the authors reflected the feedback accordingly in the final version of the questionnaire. ‘Handedness’ is also discussed in many tinnitus literatures [12, 30–32] and was evaluated to be included for the tinnitus outcome questionnaire for sound management. In the domain of ‘Tinnitus management,’ all questions were evaluated to be appropriate except one question about medication (‘*Are you currently taking any medications*?’) had a relatively low CVI of 0.78. Applying the experts’ comment of adding “due to tinnitus” to the item, the authors revised the question to ‘*Are you currently taking any medications due to your tinnitus*?’ so that the question is more specific. Lastly, the experts evaluated all items in the ‘Sound management outcome’ domain to be suitable for the tinnitus outcome questionnaire for sound management.

To the best of our knowledge, this is the first study to develop a tinnitus outcome questionnaire which includes questions specific to sound management; currently, no instruments contain questions specific to sound therapy. Content validity assessment suggested a baseline for potential questionnaire items. Numerous studies have examined the effect of sound therapy, but most of them used questionnaires that did not include specific questions about sound therapy [33, 34]. Since the tinnitus outcome questionnaire contains both tinnitus characteristics and sound management items, it would be convenient for professionals to obtain tinnitus case history and outcome information in one questionnaire and monitor any changes in tinnitus characteristics after sound therapy.

However, this study has a limitation; only the content of the questionnaire was validated. Fackrell et al. mentions that reliability, validity, responsiveness, and interpretation as key factors for validating questionnaires [29]. Further research is needed to examine internal consistency, reproducibility, and construct validity of the questionnaire as next steps. Other important concepts to consider are “responsiveness” and “minimally clinically important difference (MCID)”. Responsiveness refers to high sensitivity to any changes related to treatment and can be expressed as effect size [18, 27, 29]. Meikle et al. reviewed nine principal questionnaires (Tinnitus questionnaire, THQ, Tinnitus Severity Scale, Subjective Tinnitus Severity Scale Tinnitus Reaction Questionnaire, Tinnitus Severity Grading, Tinnitus Severity Index, THI, and Intake Interview for Tinnitus Retraining Therapy) for tinnitus and reported that they were not designed to have high “responsiveness” and lacked information about effect size [27]. Thus, additional work is necessary to develop a tinnitus outcome assessment tool with high validity, reliability, and responsiveness. MCID is also related to data interpretation: *how much change is clinically meaningful*?. Adamchic et al. examined test-rest reliability, validity, and MCID of VAS for loudness and annoyance for individuals with chronic tinnitus. It was a 16-week clinical study and the results not only showed good test-retest reliability and validity, but also showed 10–15 points of MCID estimates [35]. In spite of the study demonstrating the effectiveness of using VAS to measure tinnitus loudness and annoyance, many of the current tinnitus questionnaire do not describe MCID [18]. The tinnitus outcomes questionnaire for sound management needs to consist of MCID information, such as a grading system. Additionally, possibility of using certain tinnitus characteristics information, such as annoyance, loudness, and frequency, to create various MCID scores based on the effectiveness of sound can be explored.

Once the outcome questionnaire is developed and standardized, it would be beneficial for tinnitus patients, healthcare professionals, and researchers. As evidence-based practice is critical, establishment of such questionnaire as a counselling tool for patients would demonstrate that offered intervention or management option is capable of providing benefit to the patients. The ability to directly see intervention effects over time could lead to more motivation and engagement in tinnitus management. For healthcare professionals and researchers, various subtypes of tinnitus could be characterized and an effective intervention method could be identified for each subtype, leading to the delivery of individualized care as well as the pathway of validating tinnitus relief via sound therapy [12].
